# Integrated metabolomic and transcriptomic analyses reveal different metabolite biosynthesis profiles of *Juglans mandshurica* in shade

**DOI:** 10.3389/fpls.2022.991874

**Published:** 2022-09-26

**Authors:** Xinxin Zhang, Yuxi Li, Huiling Yan, Kewei Cai, Hanxi Li, Zhiwei Wu, Jianguo Wu, Xiangdong Yang, Haichen Jiang, Qingcheng Wang, Guanzheng Qu, Xiyang Zhao

**Affiliations:** ^1^State Key Laboratory of Tree Genetics and Breeding, Northeast Forestry University, Harbin, China; ^2^Jilin Provincial Key Laboratory of Tree and Grass Genetics and Breeding, College of Forestry and Grassland Science, Jilin Agricultural University, Changchun, China; ^3^Scientific Research Center of Harbin Forestry and Grassland Bureau, Harbin, China; ^4^Daquanzi Forest Station in Binxian County, Harbin, China

**Keywords:** *Juglans mandshurica*, light, physiology, metabolome, transcriptome

## Abstract

Light is not only a very important source of energy for the normal growth and development of plants, but also a regulator of many development and metabolic processes. The mechanism of plant growth and development under low light conditions is an important scientific question. With the promulgation of the law to stop natural forest cutting, understory regeneration is an important method for artificial forest afforestation. Here, the growth and physiological indexes of *Juglans mandshurica*, an important hardwood species in Northeast China, were measured under different shade treatments. In addition, transcriptome and metabolome were compared to analyze the molecular mechanism of shade tolerance in *J. mandshurica*. The results showed that the seedling height of the shade treatment group was significantly higher than that of the control group, and the 50% light (L50) treatment was the highest. Compared with the control group, the contents of gibberellin, abscisic acid, brassinolide, chlorophyll a, and chlorophyll b in all shade treatments were significantly higher. However, the net photosynthetic rate and water use efficiency decreased with increasing shade. Furthermore, the transcriptome identified thousands of differentially expressed genes in three samples. Using enrichment analysis, we found that most of the differentially expressed genes were enriched in photosynthesis, plant hormone signal transduction and chlorophyll synthesis pathways, and the expression levels of many genes encoding transcription factors were also changed. In addition, analysis of differentially accumulated metabolites showed that a total of 470 differential metabolites were identified, and flavonoids were the major differential metabolites of *J. mandshurica* under light stress. These results improved our understanding of the molecular mechanism and metabolite accumulation under light stress in *J. mandshurica*.

## Introduction

The renewal capacity of species in forest clusters is favorable for better inspection and management of forest ecosystems and the establishment of sustainable management strategies for ecological health forest districts in some regions (Haq et al., 2022). With the growing worldwide demand for wood products and excessive deforestation, the global forest area has been significantly reduced. According to the ninth National Forest Resources Inventory, the national forest cover rate is only 22.96%. Natural forests have more complicated structures and functions and higher biodiversity than secondary forests (Feng et al., 2014; Gaveau et al., 2016). Forest regeneration plays an important role in the succession and development of forests. Under-forest regeneration as a common regeneration method in forest regeneration can increase species diversity and richness and make the existing stands develop toward the near-natural state with different ages, mixed and stratified at the same time (Borda-Nino et al., 2021; Zhang et al., 2021a). Light intensity is one of the key environmental factors affecting the success of understory regeneration (Zhang et al., 2012). However, there are few studies on this aspect.

Light is one of the most important environmental factors controlling plant growth and development. Light is not only the main energy source of plants, but also controls various development processes in the whole life cycle of plants (Franklin, 2008). Leaves are the organs most affected by environmental conditions. The morphological and physiological changes of leaves due to light intensity changes have been confirmed in some studies (Tsubo and Walker, 2004). In shade environment, leaves display adaptations in photosynthetic structures including thinner leaves, larger leaf area (Middleton, 2001). Plants produce the carbon needed for growth and development through photosynthesis (Kirschbaum, 2004). Under forests, light is inevitably blocked by leaves and branches, which forms a sheltered light environment for lower plants (Zhang et al., 2012). The photosynthetic rate of leaves under light was higher than that of shaded leaves, but the duration was not longer than that of shaded leaves. In addition, the respiration rate of leaves under light is higher, which also leads to low total net assimilation efficiency (Lennon et al., 2021). Under shading conditions, plants regulate photosynthesis by changing enzyme activity (Raza et al., 2020), electron transport chain components (Chow and Anderson, 1987), proteins (Gao et al., 2020) and photosensitive pigments (Casal, 2013). Soybean leaves grown under shading conditions have antioxidant defense mechanisms, including the acceleration of SOD, POD, APX and CAT activities (Raza et al., 2020). Higher plants not only convert solar energy into chemical energy through photosynthesis but also use light as an information cue to control a variety of physiological reactions throughout their life cycle (Kami et al., 2010). Plants can perceive changes in the quality and quantity of light through plant hormone networks, thereby regulating their growth accordingly (Casal, 2013; Kurepin and Pharis, 2014). Under the condition of insufficient light, plants will obtain more energy by elongation of stems (Hornitschek et al., 2012). Many studies have also confirmed the occurrence of the relationship between light and plant hormone networks. The transportation and signal transduction of auxin under shade conditions are regulated (Hornitschek et al., 2012; Iglesias et al., 2018). Yi et al. found that auxin synthesis in plants increased rapidly under shade conditions (Tao et al., 2008). Abscisic acid (ABA) usually responds to various environmental stresses. Reddy et al. found that plants inhibit the occurrence of lateral buds through ABA accumulation in shade environments (Reddy et al., 2013). It can be seen that plant regulate stem elongation and inhibit branching by multiple hormones against shade tolerance.

With the development and application of multi-omics and high-throughput genome sequencing technologies, researchers have gained a clearer understanding of the molecular regulation mechanisms and metabolic pathways of plant functions, traits, and stress responses. Transcriptome sequencing is widely used because it can effectively determine gene expression profiles at the genome-wide level. In order to study the network of gene and metabolite, the combination of transcriptomics and metabolomics provides a high-throughput method to identify the function of the genes related to metabolic pathways (Qi et al., 2022). For example, molecular and metabolic insights into anthocyanin biosynthesis in *Padus virginiana* (Li et al., 2021), a study on the blue discoloration of *Raphanus sativus* (Zhang et al., 2021b), an analysis of the characterization of cold stress responses in *Nicotiana tabacum* (Jin et al., 2017), and the effects of growth environmental changes on amino acid metabolism of *Ginkgo biloba* (Guo et al., 2021) have been reported. Under both sufficient sunlight and natural shade conditions, cassava leaves were compared by transcriptome data. Ding found that shade significantly induced the expression of genes related to response in photosynthesis, light signaling, hormone-related genes and transcription factors (Ding et al., 2016). Shi ectopically overexpressed *ZmHB53* in *Arabidopsis thaliana*, suggesting that *ZmHB53* might participate in the shade response regulation of maize (Shi et al., 2019). Lyu’s study demonstrated the integration of GmGRY1-mediated signals with the gibberellin metabolic pathway in the regulation of LBL-induced shade avoidance syndrome (SAS) in soybean (Lyu et al., 2021). Although studies on shade have been reported in recent years, few studies have combined transcriptomics and metabolomics to analyze the regulatory network of the shade response.

*Juglans mandshurica*, is a plant of Julandaceae, Juglans. As one of the three hardwood tree species, together with *Fraxinus mandehurica* and *Phellodendron amurense*, it mainly grows throughout regions of northeast China. *J. mandshurica* has long been identified as a national level II rare tree species and has a high application value. *J. mandshurica* is widely used in the military industry, shipbuilding and so on due to its excellent wood properties and beautiful texture. Its fruit is rich in nutrition, has a high oil content, and has high edible value. Roots, stems and leaves of *J. mandshurica* have high health protection values, especially immature fruits. As present, more than 400 compounds have been isolated and identified, mainly including quinones, phenylpropanoids and flavonoids and so on (Luan et al., 2021). Due to the extremely high application value of *J. mandshurica*, the demand for it is increasing, so research on the sustainable development of *J. mandshurica* has become particularly important. At present, most studies focus on the chemical constituents and growth traits of *J. mandshurica* (Guo et al., 2015; Yao et al., 2015)*. J. mandshurica* is one of the main tree species of forest succession in Northeast China, and its growth and development under forest is mainly affected by light environment. Zhang et al. studied on the growth, morphology and chlorophyll content of *J. mandshurica* seedling under shade (Zhang et al., 2017). They found that *J. mandshurica* seedling under shade grows fast, leaf is broad, and the branch number is less than that under normal light. In order to adapt to shade, *J. mandshurica* seedlings have made adaptive adjustments in morphology and physiology. However, we have little knowledge about the mechanism of morphological and physiological adjustment of *J. mandshurica* in shade. Previous studies on *J. mandshurica* under shade were limited to the level of growth and physiology. In this study, we measured the growth and physiological indices of *J. mandshurica* under different shade treatments and revealed its regulatory network by combining transcriptional and metabolic analysis. By combining the analysis of growth, physiology, transcription and metabolism, we comprehensively introduced the regulation mechanism of *J. mandshurica* in shade environment for the first time. These studies have increased the researchers’ understanding of plant tolerance to shade.

## Materials and methods

### Plant materials and treatments

**Figure 1:**
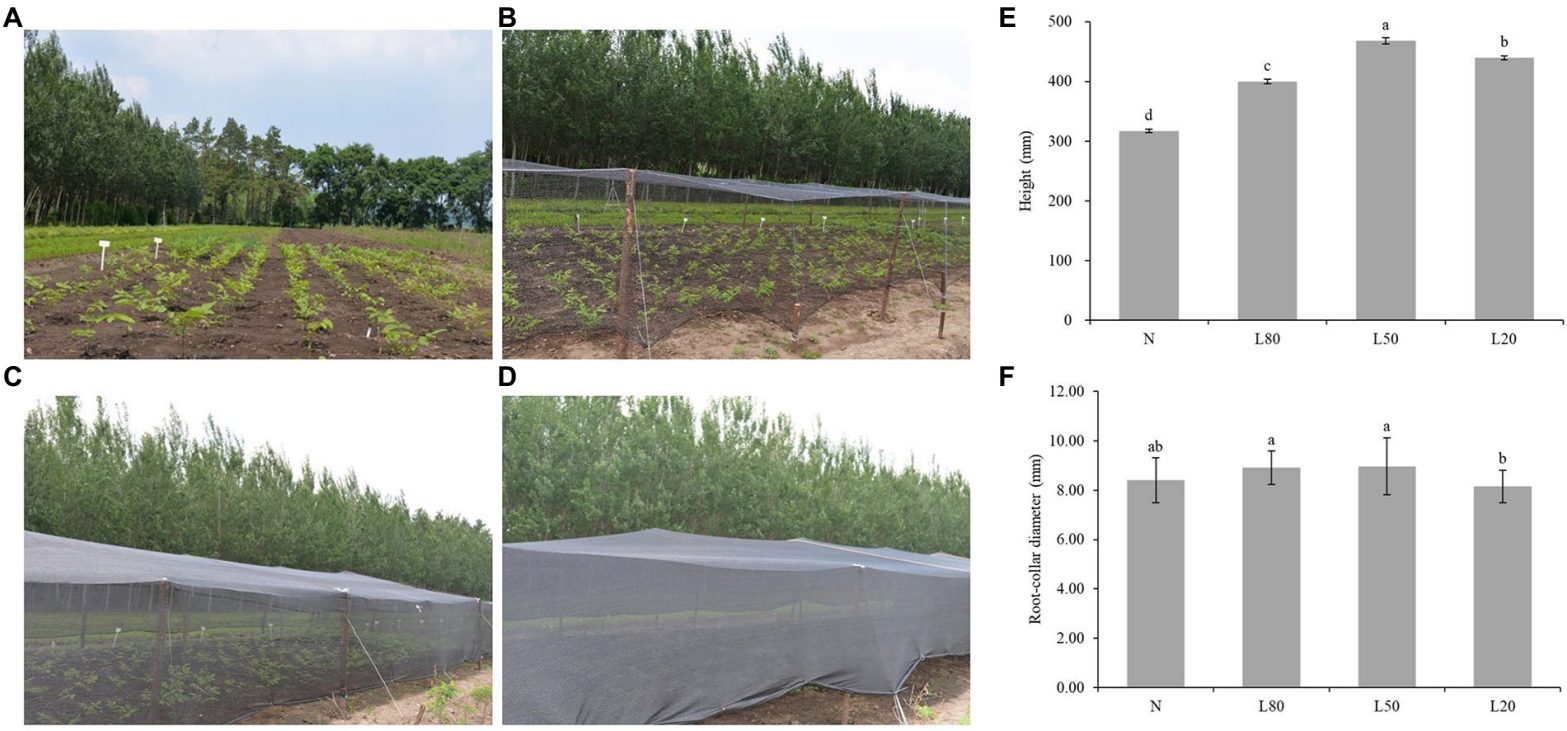
Effect of shade on the growth of *J. mandshurica*. *J. mandshurica* grown in different shade conditions, including **(A)** normal sunlight; **(B)** 80% sunlight to go through; **(C)** 50% sunlight to go through; **(D)** 20% sunlight to go through. **(E)** Height. **(F)** Root-collar diameter. N: natural sunlight. L80: 80% sunlight to go through. L50: 50% sunlight to go through. L20: 20% sunlight to go through. Error bars indicate standard deviation (SD). Different letters indicate significant differences between different treatments.

*J. mandshurica* is a native tree species in Northeast China. The study was conducted at Daquanzi Forest Station in Harbin City, Heilongjiang Province. The major geographical and climatic condition are shown in Table 1. The experimental materials were selected from local two-year-old seedlings of *J. mandshurica.* The same growth of *J. mandshurica* seedlings transplanted into an experimental field was selected in the spring of 2021. After transplanting, the seedlings were stubbled to make their growth consistent. Shading began on 11 June, 2021 and there were 50 seedlings per treatment. In order to set light, middle and heavy shade conditions respectively, we set 80% light (L80), 50% light (L50), and 20% light (L20), and the control was natural sunlight (N) according to previous studies (Wang et al., 2012; Wu et al., 2022). Different densities of shading nets were selected for different degrees of shading treatment, which were 80% light (L80), 50% light (L50), and 20% light (L20), and the control was natural sunlight (N) (Figures 1A–D). The samples were not occluded with each other. Samples were harvested after one month of shade treatment. Samples were composed of a mix of leaves from three single seedlings. Moreover, each sampled seedling provided complete and healthy leaves. All leaves were immediately frozen in liquid nitrogen and stored at −80°C.

**Table 1:** Main geographical and climatic condition of Daquanzi Farm Station.

East longitude	North longitude	Climate type	Altitude (m)	Average temperature (°C)	The annual average precipitation (mm)	Effective accumulative Temperature (°C)	Frost-free season (d)	Agrotype
127°32′47″	45°32′47″	Temperate continental climate	500	3.5	600–800	2,560	135	Dark brown soil

### Growth and leaf morphological traits measurements

After one month of shade treatment, all seedlings were measured for height and root collar diameter. Seedling height was measured by tapeline, and root collar diameter was measured by a vernier caliper. Thirty seedlings were selected to measure for replicates. The leaf area, randomly selected three seedlings (a total of 30 leaves) for measurement. The camera (CanoScan EOS 600D, Canon Inc.) was used to take pictures for leaves, and leaf length, leaf width and leaf area were measured by Image J 1.46r. Then leaf shape index was calculated by leaf shape index = leaf length/leaf width. At the same time, measured fresh weight of the 30 leaves from each treatment. Then all leaves dried at 105°C for 20 min followed by 80°C until constant weight. And the average fresh weight and dry weight of single leaf were calculated.

### Photosynthetic parameter measurements

Photosynthetic parameters were measured using the portable CIRAS-3 photosynthesis system (PP Systems, United States). It includes the net photosynthesis rate (Pn), stomatal conductance (Gs), intercellular CO_2_ concentration (Ci) and transpiration rate (Tr). Water use efficiency (WUE) was calculated by WUE = Pn/Tr. All measurements were carried out between 8:30 and 11:00 on sunny and clear days. The top third or fourth functional leaves were selected for measurement. Light was provided during sampling with ambient light in real time and the CO_2_ concentration was that of the ambient air. Other parameters were not controlled. Three replicates were made from three different leaves of the same plant and three plants were measured for each treatment.

### Plant endogenous hormone measurements

The contents of auxin (IAA), gibberellin (GA), abscisic acid (ABA), ethylene (ETH), jasmonic acid (JA), salicylic acid (SA), brassinosteroid (BR) and zeatin (ZT) were measured using Enzyme-Linked Immunosorbent Assays (ELISA). Randomly selected three healthy seedlings for mixed sampling. All samples were immediately frozen in liquid nitrogen for subsequent measurement. The methods of hormones extracted according to the description by Zhang et al. (2019a) with less modifications. The samples grinded with liquid nitrogen, then 0.5 g powder were added 5 ml 80% methanol (containing 1 mmol/l 2-tert-Butyl-4-methylphenol) and stored at 4°C to extract hormones. Absorbed the supernatant after centrifugation (5,000× *g* at 4°C, 10 min). Extracted the residues again according to the above method and merged all the supernatants together. Then supernatant was separated by solid phase extraction column, dried by nitrogen. At last, diluted into a constant volume by 2 ml sample diluents as the samples to be tested. The ELISA kit was supported by Shanghai Enzymatic Biotechnology Company Ltd. The specific operation steps were based on the instruction of manufacturer.

### Photosynthetic pigment concentrations

For photosynthetic pigments analysis, pigments were extracted by grinding leaves in 95% ethanol in the dark. For detailed steps, improved by the method of Zhang et al. (2019b). Samples mixed with random three seedlings. Fresh leaves were ground with liquid nitrogen and 0.1 g sample was dissolved in 10 ml of 95% ethanol. After standing, absorbed the supernatant and measured the absorbance values at 470, 649, 664 nm, respectively. Concentrations of photosynthetic pigments were calculated using the following equation (Zhang et al., 2019b):


$$Chl\;a=13.36\times A_{664}-5.19\times A_{649}$$

$$Chl\;b=27.43\times A_{649}-8.12\times A_{664}$$

$$\mathrm{Car}=\left(1000\times A_{470}-2.13\times Chl\;a-97.64\times Chl\;b\right)/209$$

totalChl=Chla+Chlb

where A_664_, A_649_ and A_470_ are measured absorbance at 664, 649 and 470 nm, respectively.

### Soluble sugars and starch measurements

Soluble sugar and starch contents were determined by anthrone colorimetry methods (Wei et al., 2020). All samples were ground with liquid nitrogen, and 0.1 g powder of each sample was extracted in 2 ml distilled water for 20 min in boiling water. Then centrifugation at 10000 rpm for 5 min, draw 1 ml supernatant into 50 ml tube diluted to 50 ml with distilled water. For the soluble sugars extract measurements, take 0.2 ml liquid add to 5 ml anthrone and reaction in boiling water for 10 min. Then measured absorbance at 630 nm. For the starch extract measurements, the insoluble substance was re-extracted in boiling water for 15 min. Then reaction with 1 ml perchloric acid in boiling water for another 15 min. After filtered the solution, diluted liquid to 50 ml with distilled water. Take 1 ml soluble starch and 5 ml anthrone reaction in boiling water for 10 min. Then measured absorbance at 430 nm.

### RNA isolation, library construction, and transcriptome squencing

RNA isolation, cDNA library construction and sequencing were performed following the methods described in Kai’s study (2022). Quality, concentration, and integrity of total RNA were estimated using the Qubit 2.0 Fluorimeter (Life Technologies, Carlsbad, CA, United States), Nano Photometer spectrophotometer (IMPLEM, Westlake Village, CA, United States) and an Agilent 2,100 bioanalyzer (Agilent Technologies, Santa Clara, CA, United States). PolyA-mRNA was purified with oligo (dT) magnetic beads. Fragmentation buffer was added to break the RNA into short segments, and these short segments were used as templates to synthesize first-strand cDNA using random hexamer primers. Second-strand cDNA was synthesized using DNA polymerase I. Transcriptome libraries were sequenced on an Illumina HiSeq platform (HiSeq™ 2500, United States), which generated the raw data of 150-bp paired-end reads. After filtering and removing the low-quality raw reads, the clean reads were mapped to the *J. mandshurica* reference genome using HISAT2 (Kim et al., 2015). The two shading treatments with the highest seedling height and the control were selected according to seedling height to investigate the changes in gene expression levels. We constructed nine libraries representing these three samples (N, L50 and L20) with three biological replicates.

### Gene expression analysis

The expression levels of differentially expressed genes (DEGs) were calculated based on the FPKM (fragments per kilobase of transcript per million fragments mapped). DEGs were identified using DESeq2 (Verhoeven et al., 1999; Kami et al., 2010). Genes with |log_2_Fold Change (FC)| ≥ 1 and *p* value < 0.05 were identified as DEGs. Finally, the DEGs were annotated using Gene Ontology (GO) by the GOseq R package, and KOBAS 2.0 software was used to analyze the enrichment pathways of DEGs in the Kyoto Encyclopedia of Genes and Genomes (KEGG).

### Metabolite measurements and profiling

The freeze-dried leaves were ground to a uniform powder, and 0.1 g of powder was dissolved in 1.2 ml of 70% methanol. All extracts were centrifuged at 12000 rpm for 10 min, and supernatants were filtered (PTFE, 0.22 μm). Filtered supernatants were used for measurements of broadly targeted metabolites. LC–MS/MS analysis was performed by Wuhan MetWare Biotechnology Co., Ltd., using the methods of Zhang et al. (2021b). Unsupervised principal component analysis (PCA) was performed using the statistics function in R^1^ (v.3.5.1). Normalized metabolite data were used to compare all samples. To define the differences between the samples, hierarchical cluster analysis (HCA) and orthogonal partial least-squares discriminant analysis (OPLS-DA) were calculated using R (Metabo Analyst R) (v.1.0.1). Metabolites with VIP ≥ 1 and fold change ≥2 or fold change ≤0.5 were considered differentially accumulated metabolites (DAMs). Additionally, the DAMs were mapped to KEGG pathways, and determined their significance by the *p* values on the basis of hypergeometric testing. Statistical analysis of DAMs enrichment in KEGG pathway using KOBAS 2.0 software.

### Quantitative real-time PCR (qRT-PCR) validation

To validate the transcriptome data, nine DEGs were randomly selected for qRT–PCR. All cDNA was obtained using the Prime Script RT reagent Kit with gDNA Eraser (TaKaRa, Kyoto, Japan), and qRT–PCR tests were carried out with the TaKaRa SYBR Green Mix kit (TaKaRa, Kyoto, Japan) using the ABI 7500 Fast Real-Time Detection System. The amplification system and procedure were carried out according to Li et al. (2021). Primers designed by Primer Premier 5.0 and 18S rRNA were used as the reference genes. All the primers used are listed in Supplementary Table S1. The relative expression level was calculated according to the 2^−ΔΔCT^ method.

### Statistical analysis of data

Statistical analyses were performed by SPSS version 26.0 (International Business Machines, NY, United States). Analysis of variance (ANOVA) *F* test was used to test the significance of fixed effects. One-way ANOVA was performed to test the effect of shade on all growth and physiological indices. The linear model for analysis of four treatments was as follows (Wang et al., 2018):


$$X_{ij}=\mu +T_{i}+e_{ij}$$

Where *X_ij_* is the performance of an individual tree *j* in treatment *i*; *μ* is the overall mean; *T_i_* is the fixed effect of treatment, and *e_ij_* is the random error.

## Results

### Growth and leaf morphological parameters

Higher plants have developed many strategies to adapt to a fluctuant light environment, including stem elongation. There were both significant differences (*p* < 0.01) in seedling height and root-collar diameter under different shade treatments (Supplementary Table S2). The seedling height of the shading treatment was significantly higher than that of the control group, and the L50 group had the largest growth, which was 47.73% higher than that of the N group (Figure 1E). For root-collar diameter, L50 and L80 were significantly higher than L20 (Figure 1F). However, due to the weak light of L20, the organic matter produced is mainly used for plant stem elongation, which leads to the root-collar diameter of L20 being significantly lower than that of the other groups.

Leaf area, leaf shape index, leaf fresh weight and leaf dry weight were all showed extremely significant differences (*p* < 0.01) among several treatments (Supplementary Table S2). Leaf areas of *J. mandshurica* under shade were significantly larger than control (Supplementary Figure S1A). For leaf shape index, L20 group was the minimum among all treatments (Supplementary Figure S1B). The leaf fresh weight of *J. mandshurica* under shade was significantly larger than control (Supplementary Figure S1C). And leaf fresh weight and leaf dry weight both reduced with increasing the shade level (Supplementary Figure S1D).

### Plant physiological-biochemical traits

**Figure 2:**
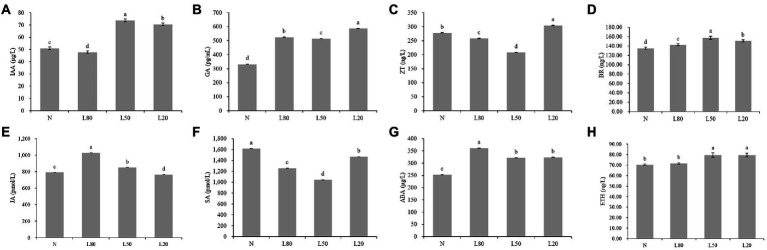
Changes in plant hormone content of *J. mandshurica* under shade. **(A)** IAA. **(B)** GA. **(C)** ZT. **(D)** BR. **(E)** JA. **(F)** SA. **(G)** ABA. **(H)** ETH. N: natural sunlight. L80: 80% sunlight to go through. L50: 50% sunlight to go through. L20: 20% sunlight to go through. The x-axis represents the samples, and the y-axis indicates the phytohormone content. Error bars indicate standard deviation (SD). Different letters indicate significant differences between different treatments.

Plant hormones are essential for plant growth and development. The levels of IAA, GA, ABA, SA, JA, ZT, ETH and Br were significantly (*p* < 0.01) expressed in different treatments (Supplementary Table S2). Compared with the control natural sunlight, the GA, ABA and BR contents of all shade treatments were significantly higher. The IAA and ETH contents of L50 and L20 were significantly higher than those of the other groups. The ZT and SA contents first decreased and then increased with increasing shade, and JA showed the opposite trend (Figure 2).

The physiological and biochemical indicators were tested, including soluble sugar and starch. For the soluble sugar and starch concentrations in different shade conditions, there were significant differences (*p* < 0.01) between different treatments (Supplementary Table S2). As well as, soluble sugar content declined with increasing the degree of shade (Supplementary Figure S2A). Furthermore, starch concentration of *J. mandshurica* in shade was significantly higher than control (Supplementary Figure S2B).

### Photosynthetic parameters and photosynthetic pigment content

**Figure 3:**
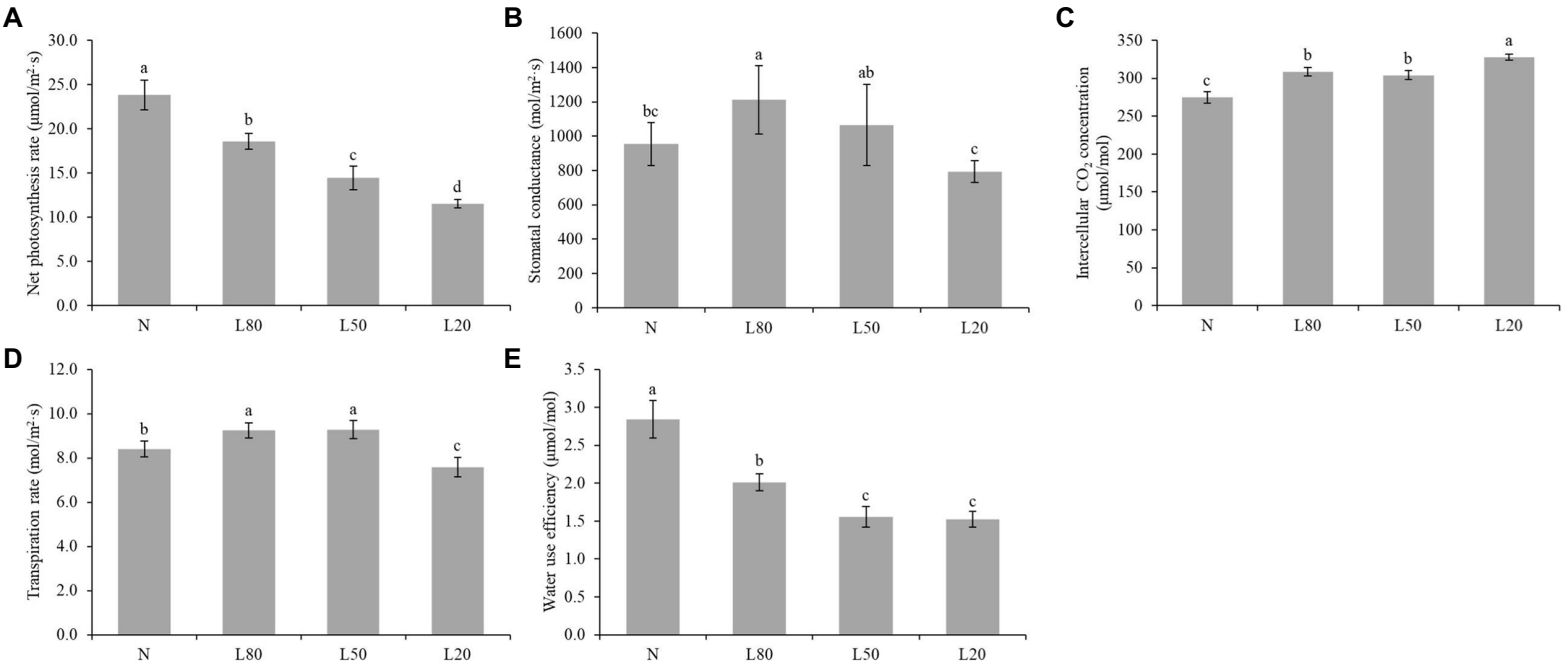
Changes in photosynthetic indicators of *J. mandshurica* under shade. **(A)** Net photosynthetic rate (Pn). **(B)** Stomatal conductance (Gs). **(C)** Intercellular CO_2_ concentration **(CI). (D)** Transpiration rate (Tr). **(E)** Water use efficiency (WUE). N: natural sunlight. L80: 80% sunlight to go through. L50: 50% sunlight to go through. L20: 20% sunlight to go through. Error bars represent the standard error. Different letters indicate significant differences between different treatments.

Among the multiple environmental factors affecting photosynthesis, light is one of the most important factors. In this study, we measured photosynthetic parameters. All photosynthetic parameters showed significant differences (*p* < 0.01) among different treatments (Supplementary Table S2). The results revealed that all shade treatments induced a significant decrease in the net photosynthesis rate (Pn) compared with the corresponding control (N), and Pn decreased gradually with increasing shading (Figure 3A). Gs of L20 was significantly lower than that of the other shade treatments (Figure 3B). Ci in the N treatment was significantly lower than that in the shade treatments (Figure 3C). Tr of L20 was significantly lower than that of the other treatments (Figure 3D). WUE decreased gradually with increasing shading (Figure 3E). Photosynthesis is an extremely complex biological process that is affected by many factors.

**Figure 4:**
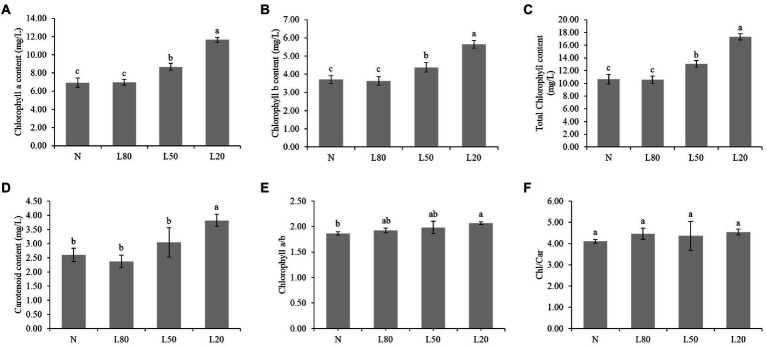
Changes in photosynthetic pigments of *J. mandshurica* under shade. **(A)** Chlorophyll a content. **(B)** Chlorophyll b content. **(C)** Total chlorophyll content. **(D)** Carotenoid content. **(E)** Chlorophyll a/b. **(F)** Chlorophyll/Carotenoid. N: natural sunlight. L80: 80% sunlight to go through. L50: 50% sunlight to go through. L20: 20% sunlight to go through. Error bars represent the standard error. Different letters indicate significant differences between different treatments.

Chlorophyll content in leaves is often used as a reference for quantitative physiological responses (Wittmann et al., 2001). The Chl a, Chl b, total chlorophyll and carotenoid content were significantly affected (*p* < 0.01) by the different treatments (Supplementary Table S2). As shown in Figures 4A–D, Chl a, Chl b, total chlorophyll and carotenoid content showed an increasing trend with increasing shade. The ratio of chlorophyll a/b was not significantly different among the different treatments (Figure 4E). The chlorophyll/carotenoid ratio showed no significant difference (Figure 4F). In this study, the shade leaves demonstrated typical pigment plasticity.

### Overview of DEGs

**Figure 5:**
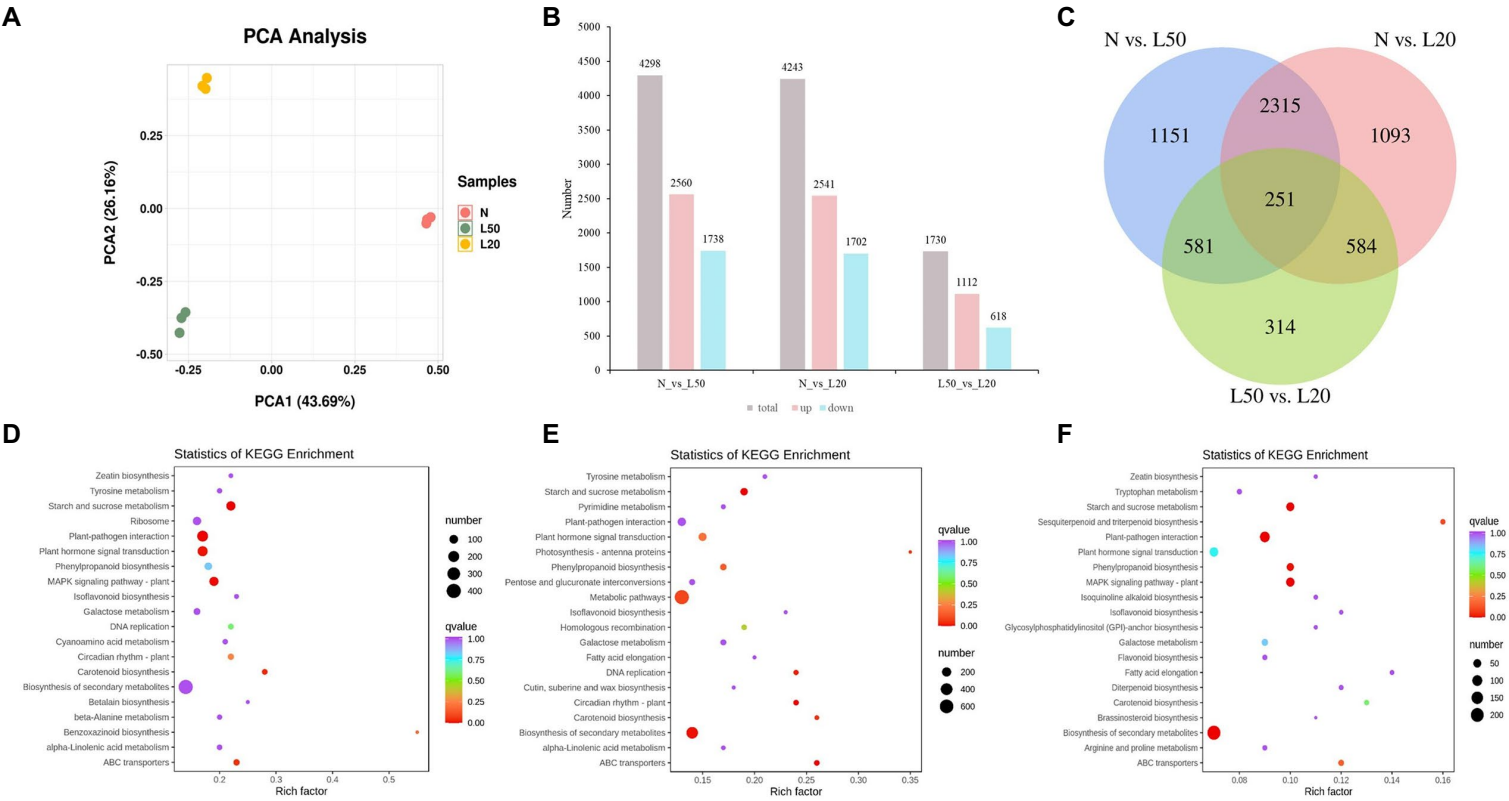
Identification and functional enrichment of differentially expressed genes of *J. mandshurica* under shade. **(A)** PCA score plot of expression profiles from different samples; **(B)** The statistics of upregulated and downregulated DEGs in different comparison groups; **(C)** Venn diagram of DEGs in different comparison groups; **(D)** The top 20 KEGG enrichment pathways of DEGs in N vs. L50. **(E)** The top 20 KEGG enrichment pathways of DEGs in N vs. L20. **(F)** The top 20 KEGG enrichment pathways of DEGs in L50 vs. L20. N: natural sunlight. L50: 50% sunlight to go through. L20: 20% sunlight to go through.

The two shading treatments with the highest seedling height and the control were selected according to seedling height to investigate the changes in gene expression levels. The *J. mandshurica* genome was used as the reference genome (Li et al., 2022). Three samples (N, L50 and L20) were used to construct nine cDNA libraries. A total of 81.75 Gb of clean data was obtained, with more than 7 Gb of clean data per sample. The average fragments scoring Q30 was higher than 90%. PCA indicated that all biological replicates were grouped together and separated well (Figure 5A). There were different genes among all comparison groups, including N vs. L50, N vs. L20, and L50 vs. L20. A total of 4,298 DEGs (2,560 upregulated and 1738 downregulated), 4,243 DEGs (2,541 upregulated and 1702 downregulated), and 1730 DEGs (1,112 upregulated and 618 downregulated) were obtained in N vs. L50, N vs. L20, and L50 vs. L20 (Figure 5B). Three comparison groups had 251 common DEGs (Figure 5C). There were more DEGs in N vs. L50 and N vs. L20 than in L50 vs. L20. This may be because the difference between L50 and L20 is small, and the difference between the two treatments is smaller than the difference between N.

### Analysis of DEGs

Photoreceptor perceiving light signals in plants leads to transcriptional regulation and developmental changes (Jiao et al., 2007). To further characterize genes affected by different shades, we performed a GO annotation system and KEGG pathway classification analysis of DEGs. All comparison groups annotated cellular components, biological processes, and molecular functions (Supplementary Figures S3A–C). The term biological process was the most common and was mainly concentrated in cellular process, metabolic process, and response to stimulus. The term molecular function was the lowest, and binding and catalytic activity were the principal terms. For cellular components, DEGs were mainly annotated in cells, cell parts, and organelles.

The top 20 KEGG pathways enriched in the differentially expressed genes are shown in Figure 5. In N vs. L50, the most enriched KEGG pathways were starch and sucrose metabolism, plant–pathogen interaction, plant hormone signal transduction, MAPK signaling pathway–plant and carotenoid biosynthesis (Figure 5D). Starch and sucrose metabolism, ABC transporters, circadian rhythm–plant, biosynthesis of secondary metabolites and DNA replication were the most enriched pathways in N vs. L20 (Figure 5E). In L50 vs. L20, the most enriched KEGG pathways were biosynthesis of secondary metabolites, plant–pathogen interaction, starch and sucrose metabolism, MAPK signaling pathway-plant and phenylpropanoid biosynthesis (Figure 5F). The starch and sucrose metabolism pathway was the top enriched KEGG pathway in all comparison groups. For the three comparison groups, the KEGG annotation analysis revealed that plant hormone signal transduction was most enriched in environmental information processing, and starch and sucrose metabolism were most enriched in metabolism along with metabolic pathways and biosynthesis of secondary metabolites (Supplementary Figures S3D–F). These data suggest that the plant hormone contents were strongly regulated by shading treatment.

### Analysis of transcription factors

**Figure 6:**
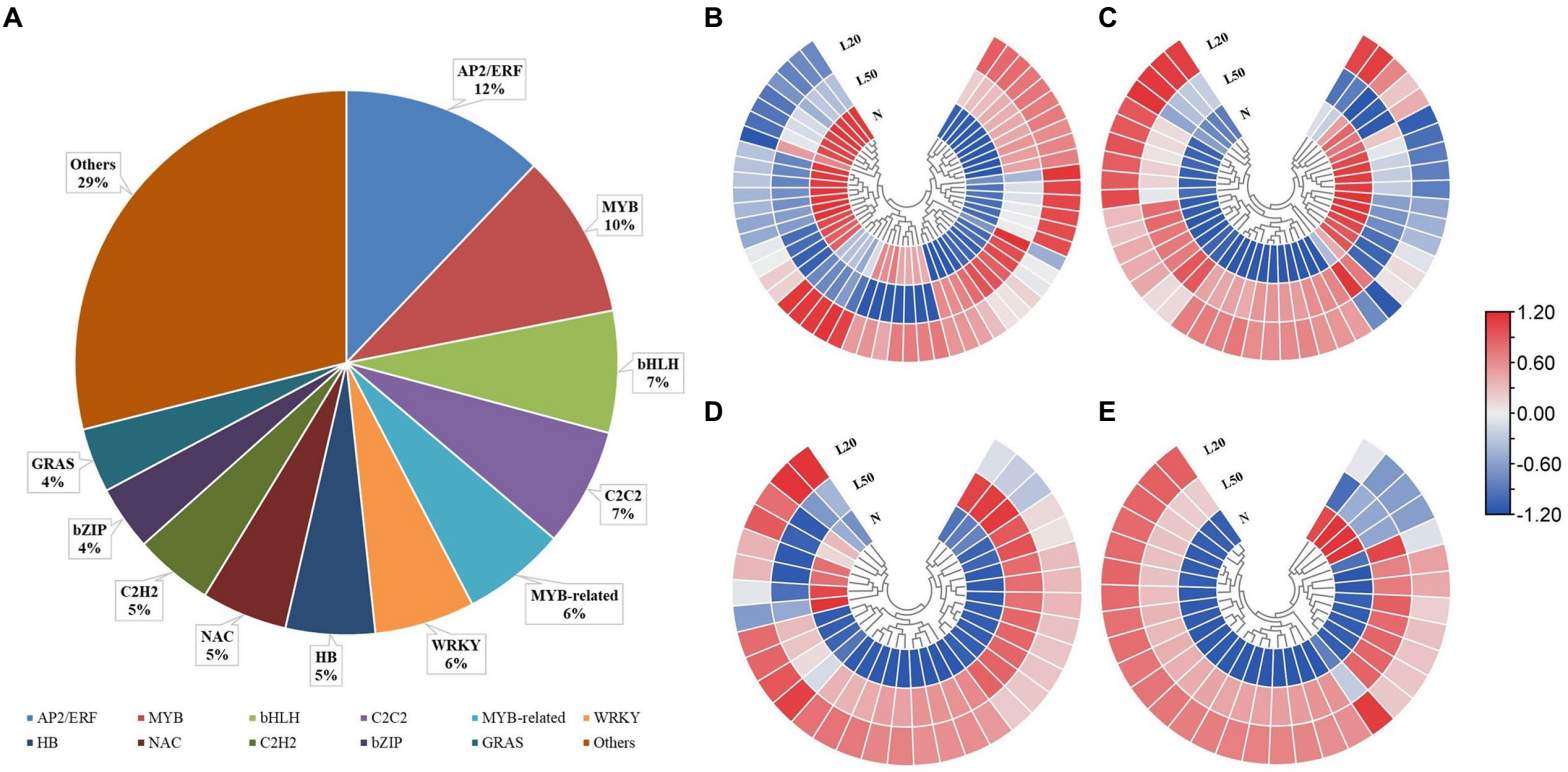
Changes in the expression levels of DEGs encoding transcription factors of *J. mandshurica* under shade. **(A)** The statistical analysis of transcription factor families in all comparison groups; Heatmap of DEGs involved in **(B)** AP2/ERF; **(C)** MYB; **(D)** bHLH and **(E)** C2C2 transcription factor family. N: natural sunlight. L50: 50% sunlight to go through. L20: 20% sunlight to go through. The color scale from blue to red indicates the expression value from low to high.

Transcription factors (TFs) play key roles in the growth and development of plants, especially in coping with various environmental stresses (Ding et al., 2016). A total of 2,643 DEGs encode 49 TF families: AP2/ERF (12%), MYB (10%), bHLH (7%), C2C2 (7%), MYB-related (6%), WRKY (6%), HB (5), NAC (5%), C2H2 (5%), bZIP (4%) and GRAS (4%) (Figure 6A). Figures 6B–E shows the expression of genes in the top four TF families. All genes of the AP2/ERF, MYB, bHLH and C2C2 families were significantly expressed in different treatments.

### Analysis of DAMs

**Figure 7:**
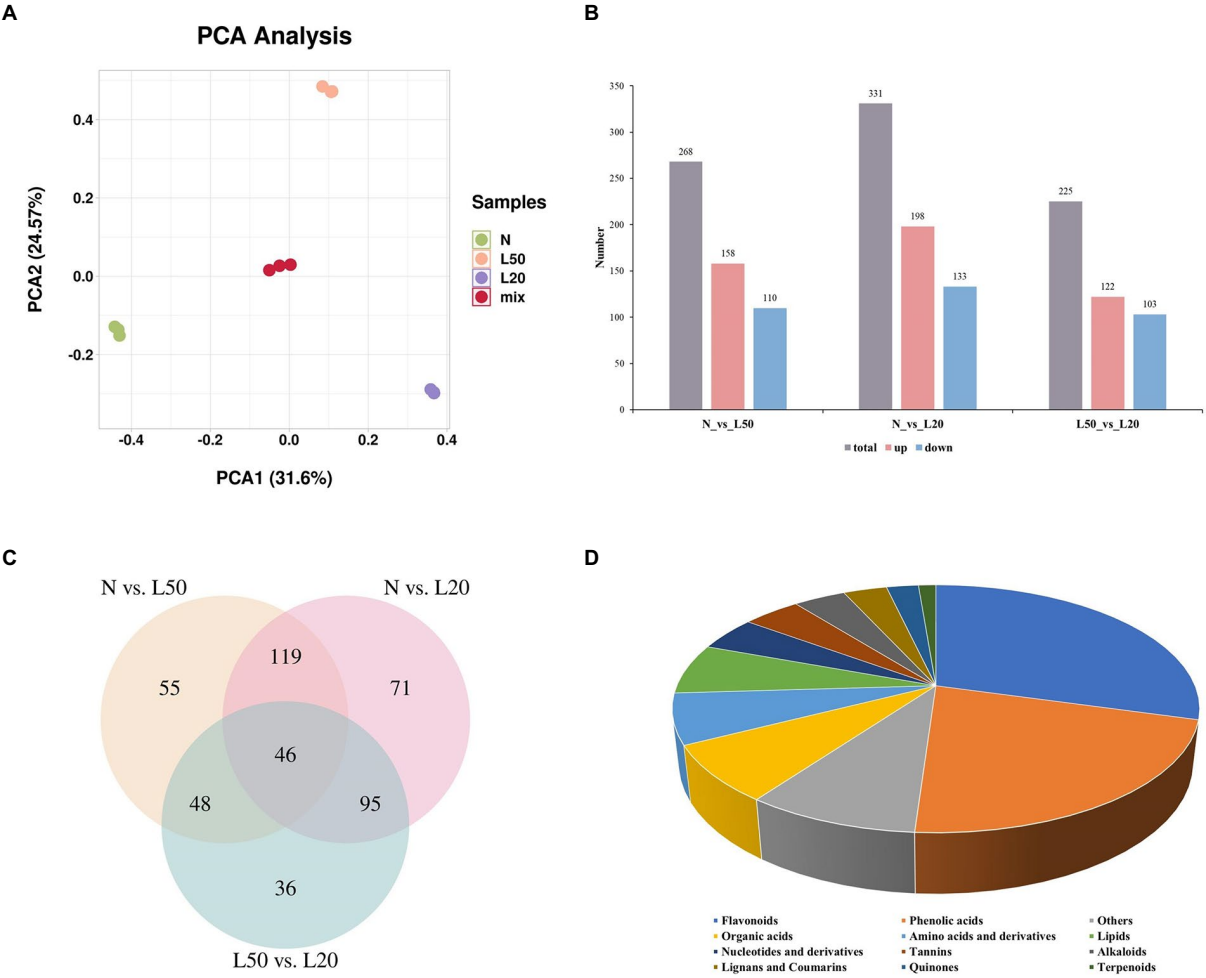
Identification of differential metabolites of *J. mandshurica* under shade. **(A)** PCA of different samples. **(B)** The statistical analysis of upregulated and downregulated differentially expressed metabolites in different comparison groups. **(C)** Venn diagram showing the number of metabolites in N, L50 and L20. **(D)** Component analysis of the identified differentially abundant metabolites from leaves. N: natural sunlight. L50: 50% sunlight to go through. L20: 20% sunlight to go through.

Study on the species and contents of metabolites in the same plant under different environments by metabolomics (Wu et al., 2022). To analyze the differences in metabolites for different shade treatments, we subjected the samples to metabolome profiling via untargeted LC–MS. PCA of N, L50, L20, and QC samples revealed effective separation (Figure 7A). A total of 268 DAMs (158 upregulated and 110 downregulated) were obtained in N vs. L50. The highest number of DAMs was observed in the N vs. L20 groups at 331 DAMs (198 upregulated and 133 downregulated). A total of 225 DAMs with 122 upregulated and 103 downregulated were identified in L50 vs. L20 (Figure 7B). The number of DAMs in the N vs. L20 groups was greater than that in the other comparison groups, and the result was similar to the DEG analysis. Three comparison groups had 46 common DAMs (Figure 7C). The top 20 enriched KEGG pathways are shown in Supplementary Figure S4. The N vs. L20 comparison contained the highest number of enriched pathways, with a total of 71.

A total of 470 DAMs were divided into 12 categories (Figure 7D), and the most abundant category was flavonoids (29%), followed by phenolic acids (22%). Interestingly, in the three comparison groups, flavonoids and phenolic acids were still the most abundant DAMs. The accumulation of flavonoids helps plants cope with various environmental stresses. High radiation is one of the most important factors affecting the dynamic changes of flavonoids. Due to the antioxidant properties of flavonoids, it can protect photosynthetic apparatus and DNA from damage caused by excessive solar irradiance (Treutter, 2005; Jiang et al., 2016).

### QRT-PCR validation of RNA-seq data

To validate the transcriptome data, nine DEGs related to photosynthesis and photosynthesis-antenna protein biosynthesis were selected for expression analysis in N, L50 and L20 by using qRT-PCR (Supplementary Figure S5). The results of the qRT-PCR analysis showed that these DEGs display similar expression patterns, as seen in the RNA-Seq analysis, indicating consistency in the RNA-Seq data obtained by Illumina sequencing and qRT-PCR.

### DEGs related to plant hormone signal transduction pathway

**Figure 8:**
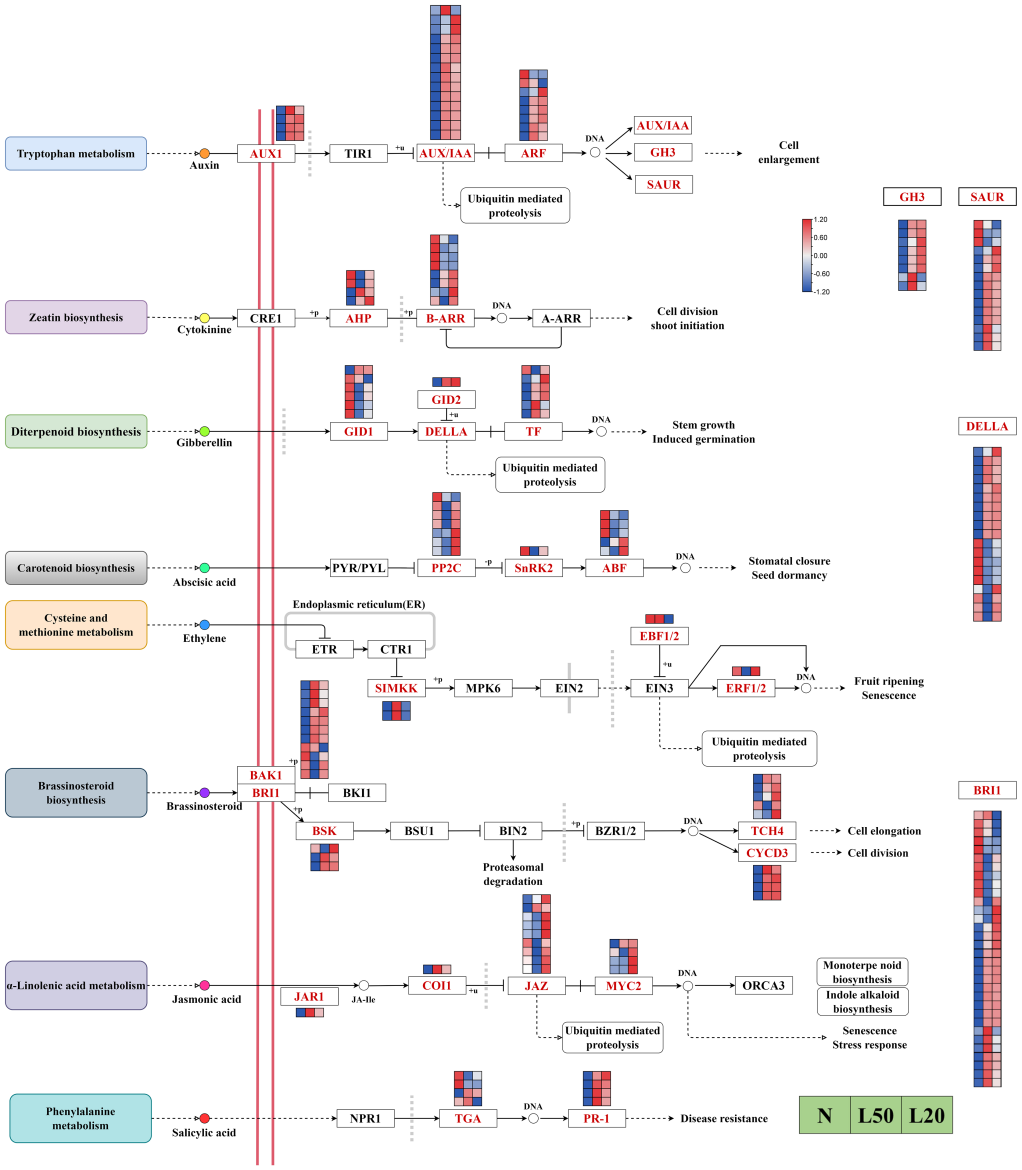
Analysis of DEGs related to phytohormone signaling pathways. The color scale from Min (blue) to Max (red) refers to the expression value from low to high. N: natural sunlight. L50: 50% sunlight to go through. L20: 20% sunlight to go through. AUX1, auxin influx carrier (AUX1 LAX family); TIR1, transport inhibitor response 1; AUX/IAA, auxin-responsive protein IAA; ARF, auxin response factor; GH3, auxin responsive GH3 gene family; SAUR, SAUR family protein; CRE1, arabidopsis histidine kinase 2/3/4 (cytokinin receptor); AHP, histidine-containing phosphotransfer protein; B-ARR, two-component response regulator ARR-B family; A-ARR, two-component response regulator ARR-A family; GID1, gibberellin receptor GID1; GID2, F-box protein GID2; DELLA, DELLA protein; TF, phytochrome-interacting factor 4; PYL/PYL, abscisic acid receptor PYR/PYL family; PP2C, protein phosphatase 2C; SNRK2, serine/threonine-protein kinase SRK2; ABF, ABA responsive element binding factor; ETR, ethylene receptor; CTR1, serine/threonine-protein kinase CTR1; SIMKK, mitogen-activated protein kinase 4/5; MPK6, mitogen-activated protein kinase 6; EIN2, ethylene-insensitive protein 2; EBF1/2, EIN3-binding F-box protein; EIN3, ethylene-insensitive protein 3; ERF1/2, ethylene-responsive transcription factor 1; BAK1, brassinosteroid insensitive 1-associated receptor kinase 1; BRI1, protein brassinosteroid insensitive 1; BKI1, BRI1 kinase inhibitor 1; BSK, BR-signaling kinase; BSU1, serine/threonine-protein phosphatase BSU1; BIN2, protein brassinosteroid insensitive 2; BZR1/2, brassinosteroid resistant 1/2; TCH4, xyloglucan: xyloglucosyl transferase TCH4; CYCD3, cyclin D3, plant; JAR1, jasmonic acid-amino synthetase; COI1, coronatine-insensitive protein 1; JAZ, jasmonate ZIM domain-containing protein; MYC2, transcription factor MYC2; ORCA3, AP2-domain DNA-binding protein ORCA2/3; NPR1, regulatory protein NPR1; TGA, transcription factor TGA; PR1, pathogenesis-related protein 1.

Multiple plant hormones participate in and coordinate shade responses in plants. The results showed a total of 188 DEGs encoding 28 enzymes in eight hormone signal transduction pathways in our study (Figure 8). In the auxin signal transduction pathway, all *AUX1* genes were upregulated under shade. All *AUX/IAA* genes, except *Jman013G0062200*, were upregulated in all shade treatments. The majority of *ARF*, *GH3* and *SAUR* genes were upregulated under shade. There were 12 DEGs encoding two enzymes in the zeatin signal transduction pathway, *AHP* genes were upregulated in L20. In the gibberellin signal transduction pathway, the majority of *TF* and *DELLA* genes were upregulated in shade. In addition, the DEG *GID2* was upregulated gradually with increasing shading degree. The DEG *SNRK2* in the abscisic signal transduction pathway was downregulated in response to shade. *Jman016G0049500,* encoding the *ABF* enzyme, was upregulated with increasing shading intensity. There were four DEGs encoding three enzymes in the ethylene signal transduction pathway, and all genes were differentially expressed in different shade conditions. In the brassinosteroid signal transduction pathway, all genes encoding *TCH4* (except *Jman005G0258000* and *Jman005G0258000*), all genes encoding *BSK* (except *Jman015G0093800*), and all genes encoding *CYCD3* were upregulated in the shade compared with the control. The majority of *BRI1* and *BAK1* genes were upregulated in shade compared with control. There were 15 genes encoding four enzymes in the jasmonic acid signal transduction pathway, and all genes except *Jman008G0050800* were upregulated in L20. In the salicylic acid signal transduction pathway, all genes encoding *PR1* were upregulated in shade, and *Jman003G0271500* and *Jman003G0271600* were downregulated in shade.

### DEGs related to photosynthesis activity

**Figure 9:**
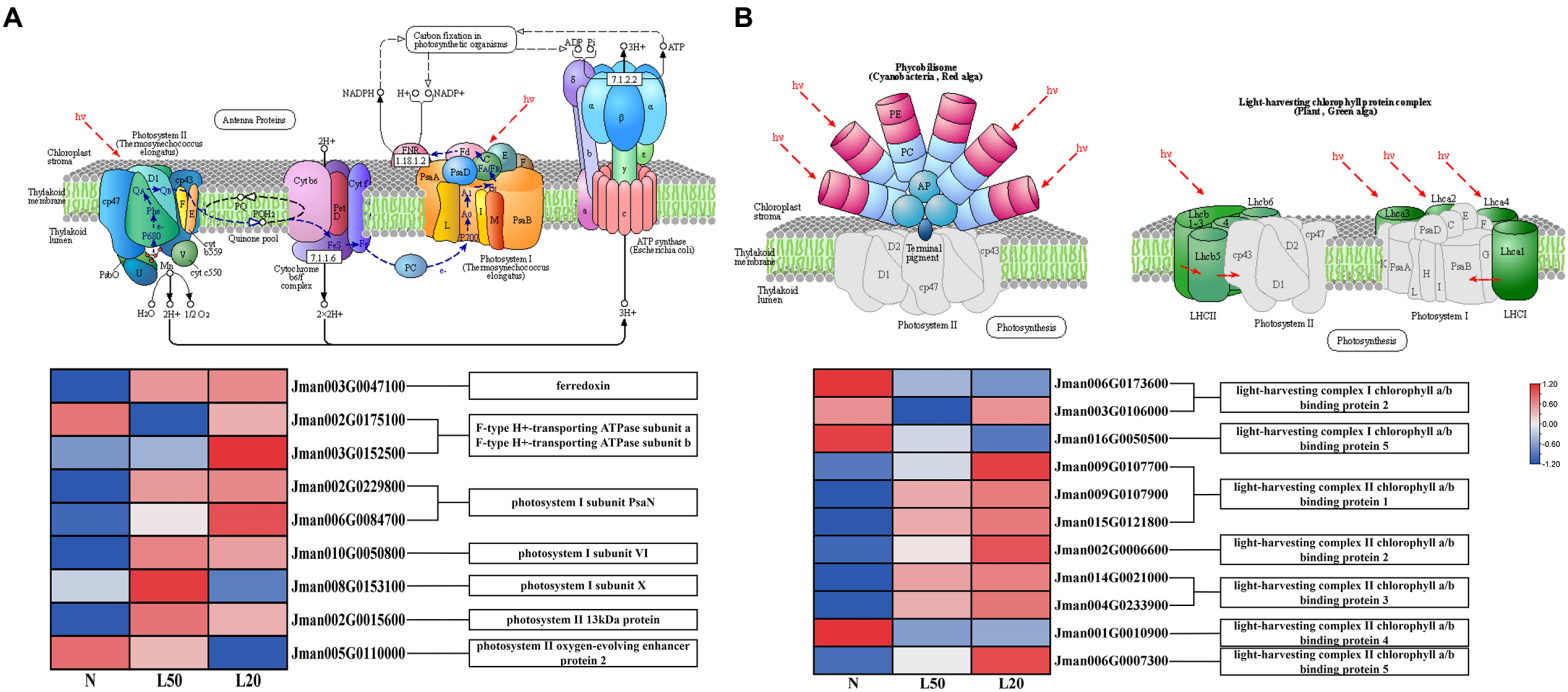
Expression of DEGs involved in response to photosynthesis. **(A)** The structure and mechanism of photosynthesis and a heatmap of the expression levels of DEGs involved in photosynthesis. **(B)** The structure of photosynthesis-antenna proteins and a heatmap of the expression levels of DEGs that were found to be involved in response to light stimuli. N: natural sunlight. L50: 50% sunlight to go through. L20: 20% sunlight to go through. The color scale from Min (blue) to Max (red) refers to the expression value from low to high.

Plant leaves use sunlight to convert energy through photosynthesis (Vass et al., 2007). Photosynthesis and photosynthesis-antenna protein biosynthesis pathways were enriched in all treatment groups. A total of 11 DEGs related to the photosynthesis process were collected under different treatments (Figure 9A). Moreover, photosynthesis I reaction center subunit-related genes *PsaN*, *PsaH* and *PsaK*, at the same time, *PsaN* (two genes) and *PsaH* (one gene) were upregulated in shade. On the other hand, there were two DEGs, *Psb28* and *PsbP*, related to photosynthesis II core complex protein: *Psb28* was upregulated and *PsbP* was downregulated in shade. Other photosynthesis-related genes included the photosynthesis electron transport gene *PetF,* which was upregulated in the shade. The F-type ATPase synthesis-related genes *atpA* and *atpB* were also differentially expressed in the shade compared with the control.

In the photosynthesis-antenna protein pathway, a total of 11 genes encoding seven enzymes related to the light-harvesting chlorophyll protein complex were differentially expressed (Figure 9B). Furthermore, the genes *Lhca2* and *Lhca5* were downregulated in the shade, and the expression of the genes *Lhcb1, Lhcb2, Lhcb3,* and *Lhcb5* increased with reduced light intensity. We have reasons to speculated that *J. mandshurica* cope with shade by regulating the photosynthesis-related genes expression.

### DEGs related to photosynthetic pigment

**Figure 10:**
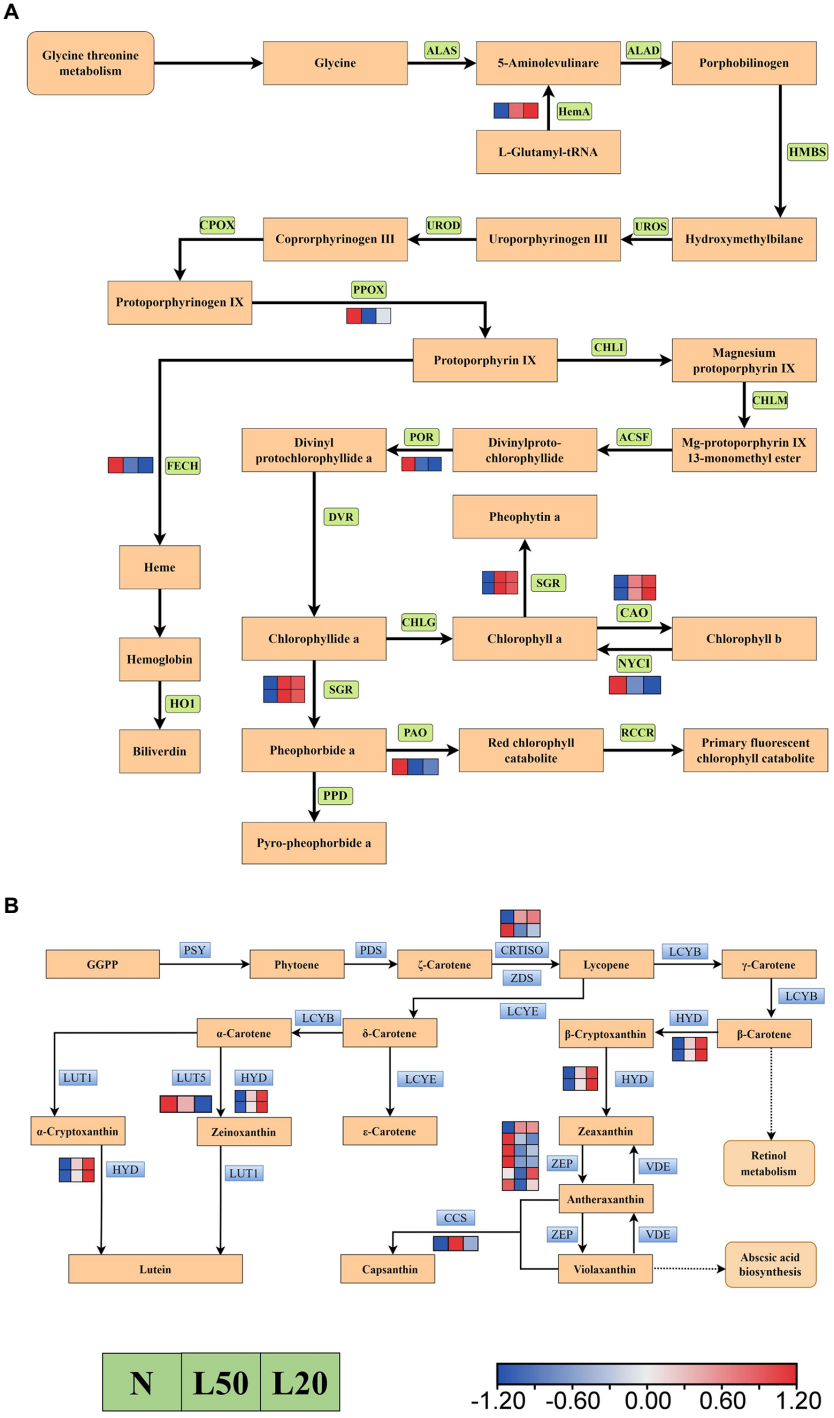
Transcriptional profiling of differentially expressed genes (DEGs) associated with pigment metabolism in *J. mandshurica* under shade. **(A)** The DEGs involved in chlorophyll metabolism. **(B)** The DEGs involved in carotenoid biosynthesis. The color scale from Min (blue) to Max (red) refers to the expression value from low to high. N: natural sunlight. L50: 50% sunlight to go through. L20: 20% sunlight to go through. **(A)** ALAS, 5-aminolevulinate synthase; HemA, glutamyl-tRNA reductase; ALAD, porphobilinogen synthase; HMBS, hydroxymethylbilane synthase; UROS, uroporphyrinogen-III synthase; UROD, uroporphyrinogen decarboxylase; CPOX, coproporphyrinogen III oxidase; PPOX, protoporphyrinogen/coproporphyrinogen III oxidase; FECH, protoporphyrin/coproporphyrin ferrochelatase; HO1, heme oxygenase 1; CHLI, magnesium chelatase; CHLM, magnesium-protoporphyrin O-methyltransferase; ACSF, magnesium-protoporphyrin IX monomethyl ester (oxidative) cyclase; POR, protochlorophyllide reductase; DVR, divinyl chlorophyllide a 8-vinyl-reductase; CHLG, chlorophyll/bacteriochlorophyll a synthase; SGR, magnesium dechelatase; CAO, chlorophyllide a oxygenase; NYC1, chlorophyll(ide) b reductase; PAO, pheophorbide a oxygenase; PPD, pheophorbidase; RCCR, red chlorophyll catabolite reductase. **(B)** PSY, 15-cis-phytoene synthase; PDS, 15-cis-phytoene desaturase; CRTISO, prolycopene isomerase; ZDS, zeta-carotene desaturase; LCYB, lycopene beta-cyclase; HYD, beta-carotene 3-hydroxylase; ZEP, zeaxanthin epoxidase; VDE, violaxanthin de-epoxidase; CCS, capsanthin/capsorubin synthase; LCYE, lycopene epsilon-cyclase; LCYB, lycopene beta-cyclase; LUT5, beta-ring hydroxylase; LUT1, carotenoid epsilon hydroxylase.

Figure 10A showed a total of 12 DEGs encoding five enzymes in the carotenoid biosynthesis pathway. Furthermore, the *HYD* and *CCS* genes were upregulated in shade. However, the *LUT5 gene* was downregulated with gradually reduced light intensity. There were two genes encoding the *CRTISO* enzyme; the *Jman010G0211300 gene* was upregulated, and the *Jman008G0248800* gene was downregulated. In the porphyrin and chlorophyll metabolism pathway, a total of 10 genes encoding eight enzymes (Figure 10B). The DEGs *NOL*, *PAO*, *POR*, *FECH* and *PPOX* were downregulated in shade, and the DEGs *CAO*, *HemA* and *SGR* were upregulated in shade.

### Flavonoid biosynthesis pathway in shade

**Figure 11:**
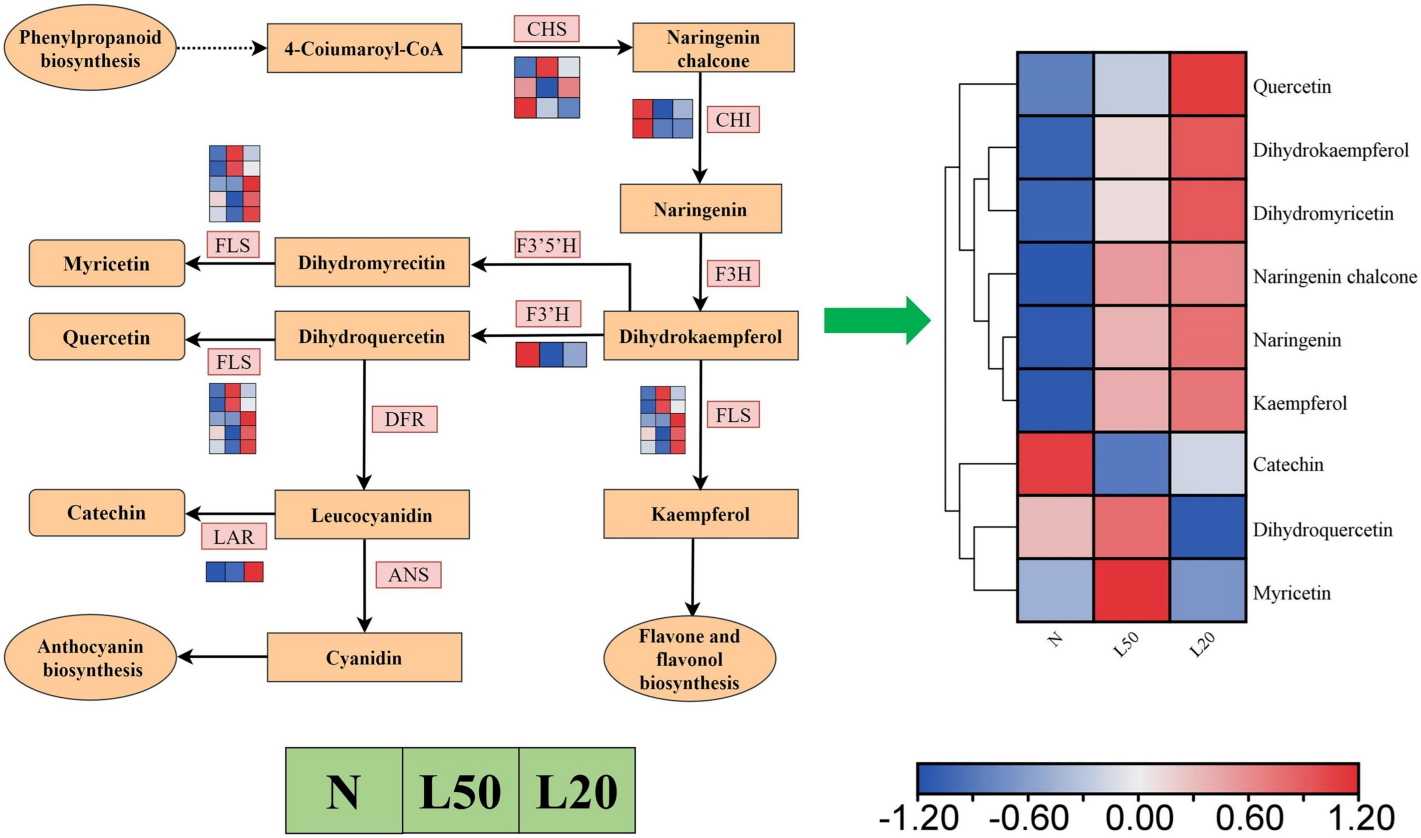
The expression of genes in the flavonoid biosynthetic pathways of *J. mandshurica* under shade. Reconstruction of the flavonoid biosynthetic pathway with the differentially expressed structural genes and the differentially accumulated metabolites (DAMs) in the flavonoid biosynthetic pathway. The color scale from Min (blue) to Max (red) refers to the metabolite contents from low to high. The cluster marker on the right side of the heatmap represents the names of each metabolite. N: natural sunlight. L50: 50% sunlight to go through. L20: 20% sunlight to go through. CHS, chalcone synthase; CHI, chalcone isomerase; F3H, naringenin 3-dioxygenase; FLS, flavonol synthase; F3’5’H, flavonoid 3′,5′-hydroxylase; F3’H, flavonoid 3′-monooxygenase; FLS, flavonol synthase; DFR, bifunctional dihydroflavonol 4-reductase/flavanone 4-reductase; ANS, anthocyanidin synthase; LAR, leucoanthocyanidin reductase.

Flavonoids were the most abundant categories in all DAMs, and the flavonoid biosynthesis pathway was enriched in all comparison groups. Figure 11 showed a total of 12 DEGs encoding five enzymes. The *LAR* gene was upregulated in shade, and the *F3’H* and *CHI* genes were downregulated in shade. The genes *CHS* and *FLS* were differentially expressed in different shade conditions, respectively. There were a total of 182 compounds of flavonoids in different treatments, and they involved seven classes (flavanones, flavanols, chalcones, flavones, flavones, flavonoid carbonoside and flavanonols). Nine metabolites accumulated differently in all comparison groups (Figure 11). The compounds quercetin, dihydrokaempferol, dihydromyricetin, naringenin chalcone, naringenin and kaempferol were upregulated with increasing shade. Other DAMs (catechin, dihydroquercetin and myricetin) were downregulated in L20.

## Discussion

### Regulation of morphological construction in shade

The development process of light regulation, including seed germination and light morphological construction of seedling, often leads to great changes in plant morphology (Jiao et al., 2007). Shade tolerance responses includes promoting the growth of stem and petiole, inhibiting the low growth and branching of leaves, and the close relationship between light and auxin signaling (Iglesias et al., 2018). In addition to *Arabidopsis* (Das et al., 2016), tomato and sunflower have also been reported to elongate hypocotyls in shade (Kurepin et al., 2007; Cagnola et al., 2012). The seedling height and leaf area of *J. mandshurica* in the shade treatments were significantly higher than that in the control treatment. However, heavy shade could affect quality of plants, and soluble sugar content declined with increasing the degree of shade. This difference in phenotype may be due to differential regulation of the expression of these hormones. Furthermore, we found that the levels of IAA, GA, ABA, SA, JA, ZT, ETH and Br were significantly expressed under different shades. Hormonal pathways are often relevant to stress. Interestingly, light induces changes in many hormonal pathways (Neff et al., 2006). Combined with the research on the application of exogenous hormones, the roles of these plant hormones in shade avoidance responses were revealed through the analysis of hormone biosynthesis and signaling transduction. Auxin regulates many aspects of plant growth and development, such as elongation growth and apical dominance, which are usually related to shade avoidance (Sawa et al., 2002). Under shade conditions, increasing IAA and GA promoted stem and petiole growth (Beall et al., 1996; Iglesias et al., 2018), and increasing ABA inhibited branching (Yang and Li, 2017). In this study, we found large DEGs in plant hormone signal transduction pathways. Auxin play a key role in the shade (Iglesias et al., 2018). Auxin response genes are mainly composed of the following three families, including *GH3* genes, *SAUR* genes and *Aux/IAA* genes (Hagen and Guilfoyle, 2002). We found that the majority of DEGs of these three families were significantly differentially expressed in shade. In addition, auxin-induced transcription factors include members of the AP2/ERF family, MYB-like family, HD-Zip superfamily and others (Nemhauser et al., 2006; Paponov et al., 2008; Chapman and Estelle, 2009). According to previous studies, AP2/ERF transcription factors directly or indirectly participate in multiple stages of plant growth and development, which control the whole process by regulating ethylene, cytosol and auxin. AP2/ERF was the most abundant TF family in our study, and the majority of DEGs were upregulated in shade. Gibberellins and auxin act as growth promoters, and auxin act as a promotor of gibberellin biosynthesis (Kurepin and Pharis, 2014). GA content increased in shade, and the majority of DEGs related to gibberellin signaling transduction were also upregulated in shade; interestingly, these DEGs were involved in plant stem growth. We speculate that the different growth among different shade treatments may be because of the different expression of multiple hormones, and auxin and GA played a key role among them. *J. mandshurica* increases stem elongation through differential expression of hormones to obtain higher stems in a low light environment. This fully explained why the seedling height of *J. mandshurica* under shading treatment was significantly higher than that under normal sunlight conditions.

### Regulation of photosynthesis in shade

Light is the driving force of photosynthesis and plays a key role as an environmental factor. Most higher plants can still grow normally under changing light conditions. Our results showed that Pn decreased with decreasing light intensity. Due to the larger leaf area could ensure that plants obtain more light energy for photosynthesis (Long et al., 2006). In shade condition, plants gain shade tolerance by increasing leaf area to get more light. In addition, different expression levels of Tr and Gs lead to different light compensation points and light saturation points. Finally, the different growth rates of *J. mandshurica* under shade were inconsistent. Light drives photosynthesis and affects multiple organs that make up photosynthesis (Vass et al., 2007). Balance of photosynthesis I and II changed due to spectral composition (Casal, 2013). There are two large protein cofactor complexes located on thylakoid membrane of photosynthetic organisms belong to photosystem I and II (Vanselow et al., 2009). Photosystem I can convert light energy into chemical energy, which is one of initial steps of photosynthesis (Amunts et al., 2010). A series of reactions such as light-induced electron transfer and water-splitting belong to oxygen-producing photosynthesis in photosystem II (Umena et al., 2011). From our transcriptomic data, a total of 11 “photosynthesis” DEGs involved in photosystems I and II, and the majority of genes were upregulated. The *psaN* gene, *Jman006G0084700*, was upregulated with increasing shade. Therefore, we speculated that it might participate in photosynthesis under shade. Under the long-term changing light intensity, higher plants adapt by changing the size of the light-harvesting antenna, which is very important. In photosynthetic organisms, the number of photosystem-related chlorophyll *a/b* protein complex (LHC) determines the size of the antenna (Tanaka and Tanaka, 2006). At the same time, 11 “photosynthesis-antenna protein” DEGs were identified, of which the majority were upregulated in this study. Photosynthetic organisms maximize photosynthetic activity under low light through gene regulation. This provided strong evidence that *J. mandshurica* have a self-regulating mechanism to maintain normal growth and development under shade.

### Regulation of photosynthetic pigments in shade

Chlorophyll is one of the most important molecules in the process of photosynthesis. It is mainly responsible for solar energy collection, charge separation and electron transport within reaction center in the photosynthetic antenna system (Tanaka and Tanaka, 2006). In this study, the Chl a, Chl b and total chlorophyll contents increased in the shade. This is consistent with the results of Dai et al. (2009). Many previous studies have shown that plants can improve light absorption efficiency by increasing pigment density per unit area to adapt to shade environment (Wittmann et al., 2001). Here, a total of 11 DEGs were identified in the porphyrin and chlorophyll metabolism pathways. For example, *HemA* plays an important role in chlorophyll synthesis, which is the initiator of chlorophyll synthesis. And glutamyl-tRNA is catalyzed to the biosynthesis of 5-aminolevulinic acid by *HemA*. The DEGs *HemA* and many key genes involved in chlorophyll a and chlorophyll b synthesis were upregulated in this study. These results are consistent with the chlorophyll content data. Carotenoids are similar to chlorophyll and transfer and convert light energy (Polívka and Frank, 2010). In the process of oxygen-evolving in green plants, the chlorophyll-carotenoid binding proteins are mainly responsible for the absorption and conversion of light energy (Green and Durnford, 1996). Carotenoids are the basic structural units of photosynthesis apparatus and the key pigment in photosynthesis besides chlorophyll. Plants have evolved complex regulatory mechanisms to cope with changes in the external environment during their long-term evolution. Green plants respond to shade environments by regulating carotenoid biosynthesis and accumulation (Lu and Li, 2008). Under different shade conditions, we found 12 DEGs in the carotenoid biosynthesis pathway, and carotenoid content was increased. The carotenoid biosynthesis pathway is linked with the plant hormones gibberellin and abscisic acid and their biosynthesis; therefore, biosynthesis and metabolism of carotenoids are regulated in multiple aspects during plant growth and development.

### Regulation of flavonoid metabolism in shade

Secondary metabolites play a key role in the plant response to environmental changes; however, there are few reports on biosynthesis plasticity of secondary metabolites (Del Valle et al., 2018). In particular, among many secondary metabolites of plants, flavonoids are very important. Flavonoids were the most abundant categories in this study, and nine metabolites accumulated differently in all comparison groups. Therefore, we further investigated the expression levels of the genes that encode important enzymes involved in flavonoid pathways. As shown in Figure 11, transcriptome analysis identified 12 DEGs involved in flavonoid pathways. For example, some key genes in flavonoid pathways, including *CHS*, *CHI*, *FLS*, *LAR* and *F3’H*, were significantly differentially expressed in the different treatment groups. In this study, the dynamic changes of flavonoid accumulation in *J. mandshurica* were similar to previous reports (Wang et al., 2012). Flavonoid biosynthesis is regulated by MYB, which directs flavonoid accumulation by controlling a set of structural genes (Lai et al., 2013; Yan et al., 2021). Meanwhile, flavonoid biosynthesis is regulated by bHLH (Li et al., 2021). In our study, the analysis of the data revealed that MYB and bHLH were the most abundant transcription factor family, except AP2/ERF. In particular, a total of 28 MYB genes and 27 bHLH genes were upregulated in shade. MYB and bHLH TFs display different expression under different shade, consistent with other studies, they involved in regulating flavonoid biosynthesis (Cominelli et al., 2008; Afrin et al., 2014).

We all know that UDP-glycosyltransferase can catalyze glycosylation, and O-methyltransferase can catalyze the information of O-methylated flavonoids, which play crucial roles in the biosynthesis of flavonoids (Li et al., 2018). In our study, the expression of some genes encoding UDP-glycosyltransferase and O-methyltransferase were different under light stress. The results suggested that these UDP-glycosyltransferase and O-methyltransferase genes may play important roles in the variation in flavonoid metabolites. In addition, some studies have also shown that flavonoids have potentially protective effects against environmental stresses. Increasing flavonoid levels in plants can increase tolerance to cold and salt stress (Xu et al., 2020; Schulz et al., 2021). Flavonoids have been suggested to serve multiple functions in photoprotection (Agati and Tattini, 2010). Schulz found that high flavonoid content in *Arabidopsis* showed awfully high tolerance to UV-B (Schulz et al., 2021). Furthermore, there are reports showed that flavonoid accumulations varied with the degree and duration of stress (Fini et al., 2011). It can be seen that the flavonoid accumulations can enhance shade tolerance in light stress. Therefore, flavonoids play an extremely key role in light stress response of *J. mandshurica*.

## Conclusion

In this study, *J. mandshurica* under different shading conditions were used as the research materials to explore the similarities and differences between their growth indices, physiological indices, molecular mechanisms and metabolites. *J. mandshurica* showed higher seedling height and larger leaf area under shade conditions. In physiological indicators, plant hormone content, photosynthetic indicators and photosynthetic pigments of *J. mandshurica* were significantly different in different shade conditions. By transcriptomic analyses, we found that most of the differentially expressed genes were enriched in plant hormone signal transduction, photosynthesis, chlorophyll and carotenoid biosynthesis pathway. By analysis of metabolomic, flavonoids were the major differential metabolites of *J. mandshurica* under light stress. In summary, *J. mandshurica* adapts to the shaded environment through its own various regulations. The shading environment led to the stem elongation of *J. mandshurica*, and insufficient light under the forest can promote the height growth of *J. mandshurica* faster, which could complete the forest regeneration better and faster. Moreover, the analysis of transcriptome and metabolome further increased our understanding of the molecular mechanism under shading conditions and provided a basis for further research. However, the environment under the forest is more complex, and the regeneration of *J. mandshurica* under forest requires further investigation.

## Data availability statement

The datasets presented in this study can be found in online repositories. The names of the repository/repositories and accession number(s) can be found at: https://www.ncbi.nlm.nih.gov/, PRJNA848847.

## Author contributions

XZ, YL and HY was a major contributor in writing the manuscript. ZW, JW, XY and HJ worked on plant sample collection and measurement. KC and HL contributed to genome annotation and transcriptome analysis. QW conceived the study. XZ participated in its design and data interpretation. GQ revised the manuscript critically. All authors contributed to the article and approved the submitted version.

## Funding

This study was supported by the Fundamental Research Funds for the Central Universities (No. 2572020DR04).

## Conflict of interest

The authors declare that there is no commercial or financial relationships in the study that may be considered a potential conflict of interest.

## Publisher’s note

All claims expressed in this article are solely those of the authors and do not necessarily represent those of their affiliated organizations, or those of the publisher, the editors and the reviewers. Any product that may be evaluated in this article, or claim that may be made by its manufacturer, is not guaranteed or endorsed by the publisher.

## Footnotes

^1^ www.r-project.org/
