# Supplementary material for: Integrated metabolomic and transcriptomic analyses reveal different metabolite biosynthesis profiles of Juglans mandshurica in shade
Source: Front Plant Sci. 2022 Sep 26;13:991874. doi: 10.3389/fpls.2022.991874 (PMC9552962; doi:10.3389/fpls.2022.991874)
Supplement: Supplementary file 1 [file Data_Sheet_1.zip › Supplementary Material.docx]

Supplementary Material

# Supplementary Figures and Tables

## Supplementary Figures


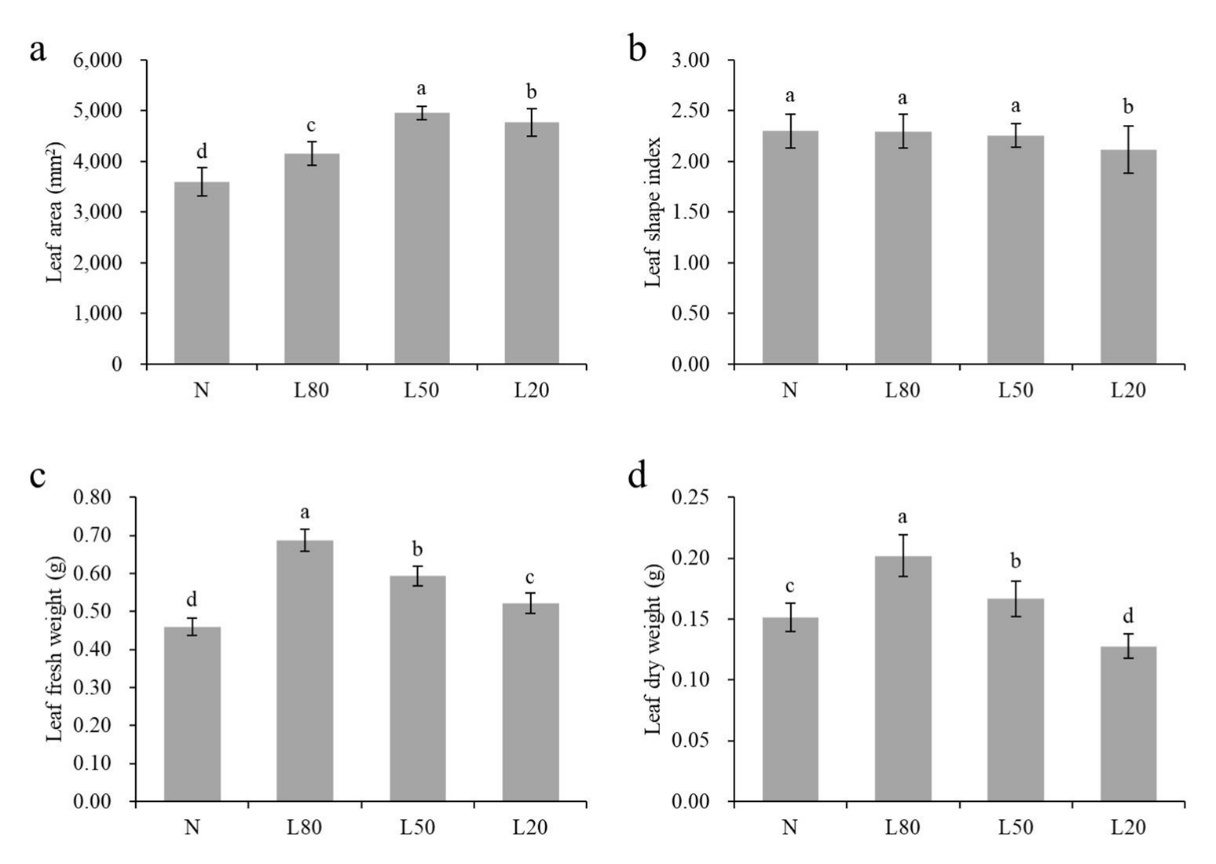


**Supplementary Figure S1.** Changes in leaf morphological parameters of *J. mandshurica* under shade. (a) Leaf area. (b) Leaf shape index. (c) Leaf fresh weight. (d) Leaf dry weight. N: natural sunlight. L80: 80% sunlight to go through. L50: 50% sunlight to go through. L20: 20% sunlight to go through. The error bars represent the standard error. Different letters indicate significant differences between different treatments.


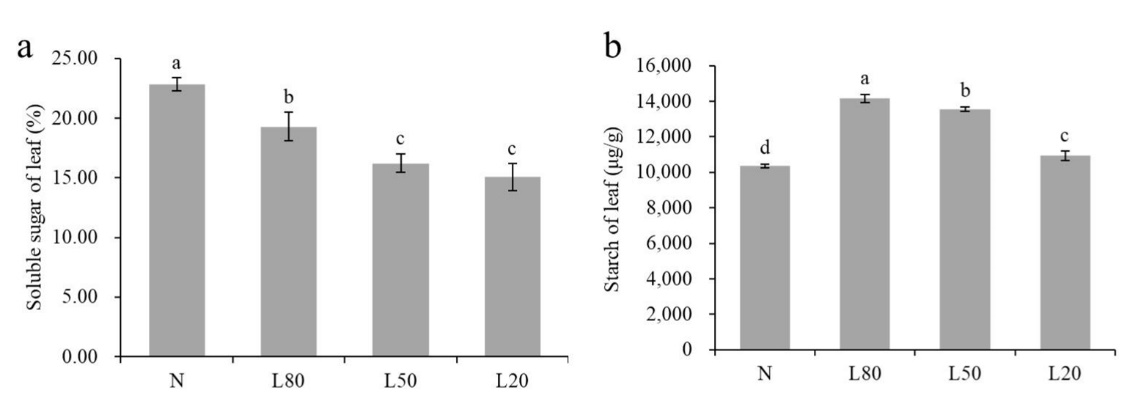


**Supplementary Figure S2.** Changes in leaf morphological parameters of *J. mandshurica* under shade. (a) Leaf area. (b) Leaf shape index. (c) Leaf fresh weight. (d) Leaf dry weight. N: natural sunlight. L80: 80% sunlight to go through. L50: 50% sunlight to go through. L20: 20% sunlight to go through. The error bars represent the standard error. Different letters indicate significant differences between different treatments.


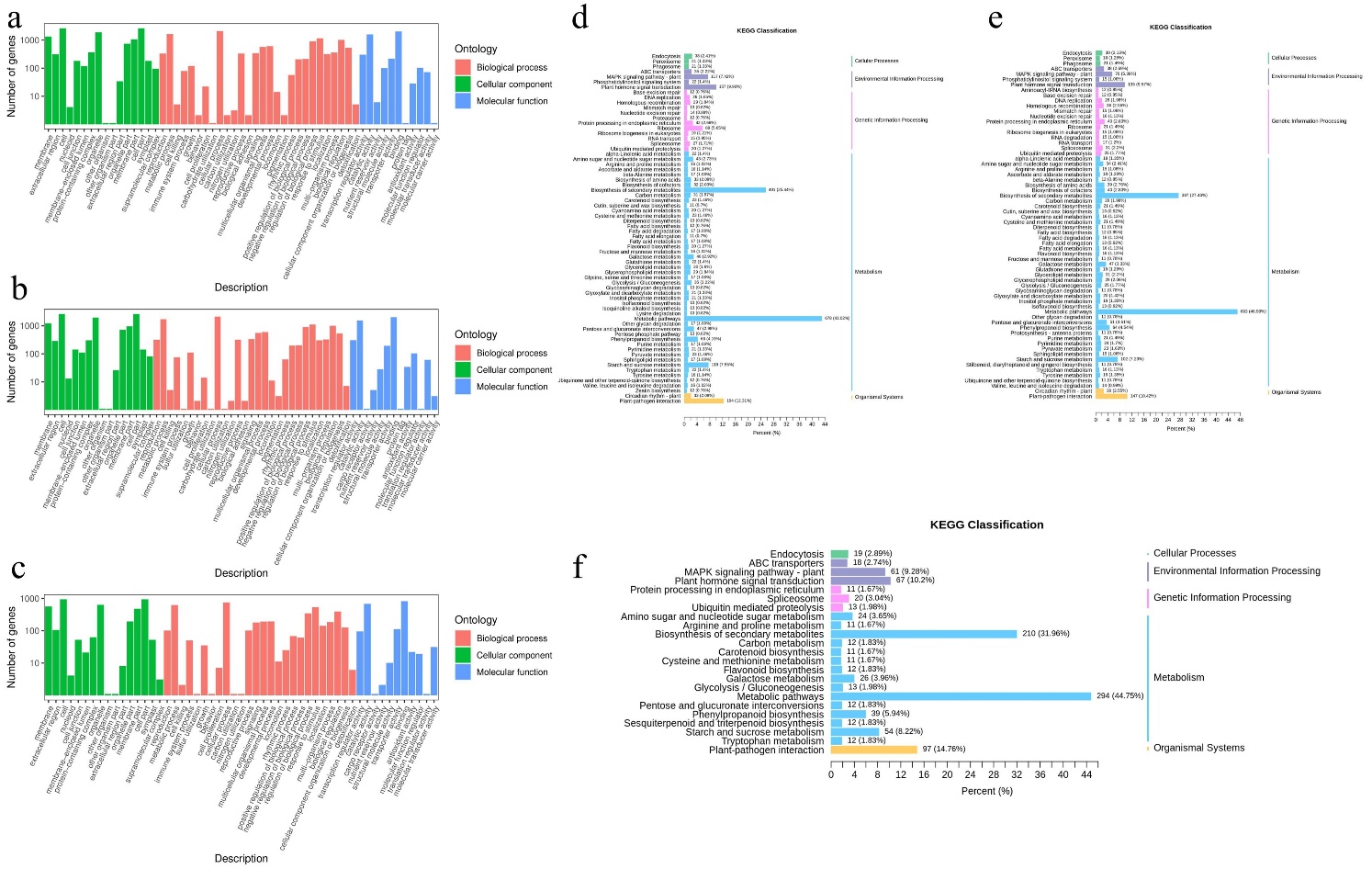


**Supplementary Figure S3.** GO classification of differentially expressed genes in the *P. koraiensis* under light stress. (a) The GO classification of DEGs in N vs. L50. (b) The GO classification of DEGs in N vs. L20. (c) The GO classification of DEGs in L50 vs. L20. (d) The KEGG classification of DEGs in N vs. L50. (e) The KEGG classification of DEGs in N vs. L20. (f) The KEGG classification of DEGs in L50 vs. L20. N: natural sunlight. L50: 50% sunlight to go through. L20: 20% sunlight to go through.


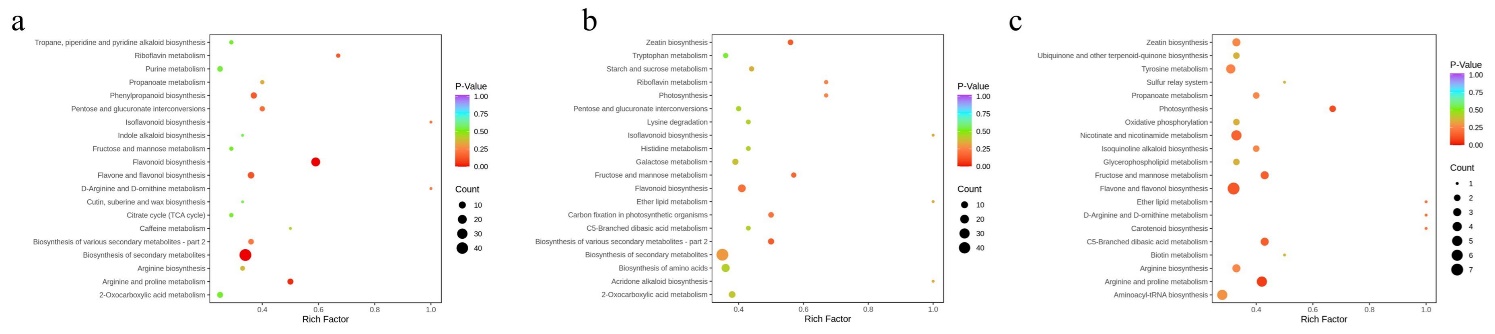


**Supplementary Figure S4.** (a) The top 20 KEGG enrichment pathways of differentially accumulated metabolites (DAMs) in N vs. L50; (b) The top 20 KEGG enrichment pathways of DAMs in N vs. L20; (c) The top 20 KEGG enrichment pathways of DAMs in L50 vs. L20. N: natural sunlight. L50: 50% sunlight to go through. L20: 20% sunlight to go through.


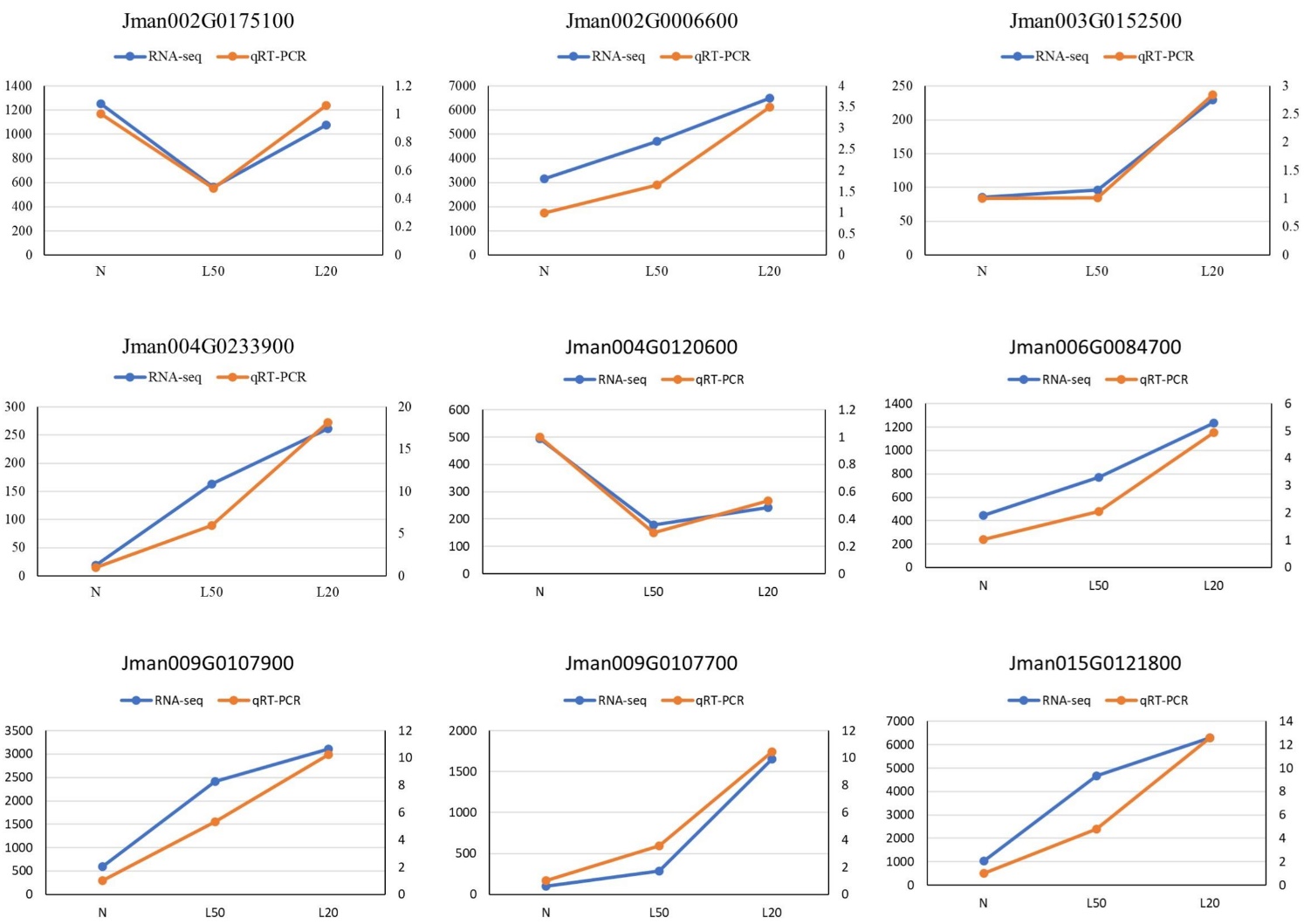


**Supplementary Figure S5.** RT–qPCR verification of the expression levels of 9 DEGs identified by RNA sequencing. The X-axis represents the different samples. The Y-axis on the left represents the FPKM value obtained by RNA-Seq, while the Y-axis on the right indicates the relative gene expression levels analyzed by qRT–PCR. N: natural sunlight. L50: 50% sunlight to go through. L20: 20% sunlight to go through.

## Supplementary Table

**Supplementary Table S1** Specific primers used for qRT-PCR analysis.

**Supplementary Table S2** ANOVA of multiple traits for *J. mandshurica.*

**Supplementary Table S3** The top 20 KEGG enrichment pathways of differentially expressed genes in N VS. L20.

**Supplementary Table S4** The top 20 KEGG enrichment pathways of differentially expressed genes in L50 VS. L20.

**Supplementary Table S5** Statistics of the numbers of transcription factor.

**Supplementary Table S6** Statistics of differentially expressed genes encoding transcription factors.

**Supplementary Table S7** Statistics of differentially expressed genes involved in plant hormone signal transduction.

**Supplementary Table S8** Statistics of differentially expressed genes involved in photosynthesis.

**Supplementary Table S8** Statistics of differentially expressed genes involved in photosynthesis - antenna protein.

**Supplementary Table S10** Statistics of differentially expressed genes involved in porphyrin and chlorophyll metabolism.

**Supplementary Table S11** Statistics of differentially expressed genes involved in carotenoid biosynthesis.

**Supplementary Table S12** Statistics of differentially expressed genes involved in flavonoid biosynthesis.

**Supplementary Table S13** The top 20 KEGG enrichment pathways of differentially expressed genes in N VS. L50.

**Supplementary Table S14** Sample sequencing data evaluation statistics.

**Supplementary Table S15** GO functional annotation of differentially expressed genes (DEGs).

**Supplementary Table S16** The list of 470 differential accumulation metabolites detected among different shade conditions.
